# Strengthening policy engagement when scaling up interventions targeting non-communicable diseases: insights from a qualitative study across 20 countries

**DOI:** 10.1093/heapol/czae043

**Published:** 2024-11-18

**Authors:** Anusha Ramani-Chander, Amanda G Thrift, Josefien van Olmen, Edwin Wouters, Peter Delobelle, Rajesh Vedanthan, J Jaime Miranda, Jan-Walter De Neve, Maria Eugenia Esandi, Jaap Koot, Dike Ojji, Zulma Ortiz, Stephen R Sherwood, Helena Teede, Rohina Joshi

**Affiliations:** Department of Medicine, School of Clinical Sciences at Monash Health, Monash University, Melbourne 3168, Australia; Monash Centre for Health Research and Implementation, Monash University and Monash Health, Clayton, Melbourne 3168, Australia; Department of Medicine, School of Clinical Sciences at Monash Health, Monash University, Melbourne 3168, Australia; Department of Family Health and Population Medicine, University of Antwerp, Antwerp 2000, Belgium; Department of Sociology, Centre for Population, Family & Health, Faculty of Social Sciences, University of Antwerp, Antwerp 2000, Belgium; Chronic Diseases Initiative for Africa, University of Cape Town, Rondebosch 7925, South Africa; Department of Public Health, Vrije Universiteit Brussel, Brussels 1090, Belgium; Department of Population Health, NYU Grossman School of Medicine, New York 10016, USA; CRONICAS Center of Excellence in Chronic Diseases, Universidad Peruana Cayetano Heredia, Lima 15074, Peru; Sydney School of Public Health, Faculty of Medicine and Health, University of Sydney, Sydney 2050, Australia; Heidelberg Institute of Global Health, Faculty of Medicine and University Hospital, Heidelberg University, Heidelberg 69120, Germany; Department of Economy, Universidad Nacional del Sur, Bahía Blanca B8001LBD, Argentina; Faculty of Medical Sciences, National University of Cuyo, Fundacion Huesped, Mendoza, Buenos Aires C1427CEA, Argentina; Unit of Global Health, Department of Health Sciences, University of Groningen, University Medical Center Groningen, Groningen 9700 RB, The Netherlands; Department of Internal Medicine, Faculty of Clinical Sciences, University of Abuja and University of Abuja Teaching Hospital, Gwagwalada, Abuja 228, Nigeria; Faculty of Medical Sciences, National University of Cuyo, Fundacion Huesped, Mendoza, Buenos Aires C1427CEA, Argentina; Fundación EkoRural, Quito 170904, Ecuador; Knowledge, Technology and Innovation, Wageningen University, Wageningen 6700, The Netherlands; Monash Centre for Health Research and Implementation, Monash University and Monash Health, Clayton, Melbourne 3168, Australia; School of Population Health, University of New South Wales, Sydney 2052, Australia; The George Institute for Global Health, New Delhi 110025, India

**Keywords:** Implementation, non-communicable diseases, policy, qualitative research, developing countries, public health

## Abstract

Policy engagement is an essential component of implementation research for scaling up interventions targeting non-communicable diseases (NCDs). It refers to the many ways that research team members, implementers and policymakers, who represent government decision-making, connect and interact to explore common interests. Well-conducted engagement activities foster co-production, local contextualization and assist in the successful translation of research evidence into policy and practice. We aimed to identify the challenges and facilitators to policy engagement during the early implementation phase of scale-up research studies. This qualitative study was focused on the research projects that were funded through the Global Alliance for Chronic Diseases in the 2019 round. Nineteen project teams opted to participate, with these studies implemented in 20 countries. Forty-three semi-structured stakeholder interviews, representing research, implementation and government were undertaken between August 2020 and July 2021. Transcripts were open-coded using thematic analysis to extract 63 codes which generated 15 themes reflecting both challenges and facilitators to undertaking policy engagement. Knowledge of the local government structures and trusting relationships provided the foundation for successful engagement and were strengthened by the research. Four cross-cutting concepts for engagement were identified and included: (1) the importance of understanding the policy landscape; (2) facilitating a network of suitable policy champions, (3) providing an environment for policy leaders to genuinely contribute to co-creation and (4) promoting two-way learning during researcher–policymaker engagement. We recommend undertaking formative policy analysis to gain a strategic understanding of the policy landscape and develop targeted engagement plans. Through engagement, researchers must facilitate cohesive vision and build a team of policy champions to advocate NCD research within their networks and spheres of influence. Ensuring equitable partnerships is essential for enabling local ownership and leadership. Further, engagement efforts must create a synergistic policymaker–researcher lens to promote the uptake of evidence into policy.

Key messages

- Engagement with policymakers is necessary for co-production, local contextualization and securing commitments for implementation and scale-up, a global health priority in the sustainable development goals era.
- This study has provided practical insights by collating the challenges to policy engagement from across 19 different studies. The collated suggestions and strategies identified provide guidance on how the policy engagement process can be better planned and supported including the need for formative policy analysis.
- The lessons on the practical realities of undertaking policy engagement for research studies provide important insights for researchers when planning such activities during implementation and scale-up of interventions.

## Introduction

Scaling up evidence-based interventions for non-communicable diseases (NCDs) is a global health priority, crucial for enhancing the health and well-being of populations (Bukhman *et al*., 2015; World Health Organization, 2020; Herrick and Reubi, 2021). Despite the spotlight being placed on NCDs, they often remain under prioritized during policy-making at the country-level, especially in resource poor settings (Roth *et al*., 2020).

Implementation research provides evidence on how to shape the scalability of complex health interventions and helps to identify contextually relevant strategies for making successful policy and practice changes (Kruk *et al*., 2016; Marten *et al*., 2021). Such evidence is valuable to policymakers, but there are several challenges to taking up this evidence to support health policy decision-making (Oliver *et al*., 2014). Persistent problems at the government and health system level, such as competing health priorities and limited resource allocation for NCD risk factors such as hypertension and diabetes, which are often silent killers, makes scaling up of NCD-related interventions particularly challenging.

Policy engagement is an ‘umbrella term describing the many ways that researchers and policymakers connect and explore common interests at various stages in their respective research and policy making processes’ (University of Oxford). Thoughtfully planned and executed engagement promotes equitable and sustainable translation of evidence-based research into policy and practice (Labrique *et al*., 2018; Lazo-Porras *et al*., 2020; Murphy, 2022; Akter *et al*., 2023).

Presently, there is a limited understanding of how research team members engage with policymakers to secure buy-in and long-term commitments for the scale-up of NCD-related interventions, particularly in low- and middle-income countries (LMICs). A better understanding of the practical dynamics of policy engagement activities will offer valuable insights into the intricate interplay and connections that occur in the intersections of various disciplines such as research and policy (Sheikh *et al*., 2016). This information can assist researchers to strategically plan issues such as optimal time for sharing research findings, identifying political windows, developing appropriate skill sets for teams and participation/representation in policy decisions (Jagosh *et al*., 2015; Howlett *et al*., 2017). Overall improving how implementation researchers can effectively support the scale-up process (Collins *et al*., 2022; Jackson-Morris *et al*., 2022).

In 2019, the Global Alliance for Chronic Diseases (2019) (GACD) funded 27 research projects, with an investment of ∼ USD 50 000 000 (Global Alliance for Chronic Diseases, 2019a) to improve the evidence base for scaling up interventions to prevent and/or control hypertension and/or diabetes in vulnerable settings (see Supplementary Appendix 1 for list of funded projects). Early and extensive policy engagement was a prerequisite of the planned research (Global Alliance for Chronic Diseases, 2018).

We examined the experiences of stakeholders, funded through this GACD initiative (Global Alliance for Chronic Diseases, 2019b), to understand how policy engagement occurs during the early implementation phase of these studies. We sought to answer the following research questions through the collective examination of these studies:

1. What challenges were encountered when undertaking policy engagement activities during the early implementation phase of scale-up research studies?
2. Which strategies and activities facilitated the policy engagement process?

The projects were awarded pre-pandemic, but the early implementation occurred during the pandemic. This study helped to capture how policy engagement unfurled when priorities of governments and policymakers shifted from NCD research to pandemic response.

## Methods

Please see Supplementary Appendix 2: Detailed Methods for further details.

### Study setting

This study forms part of the joint research activities undertaken by the GACD Upscaling Working Group Collaboration, the protocol for which has been described previously (Global Alliance for Chronic Diseases, 2019b; Ramani-Chander *et al*., 2022). One of the GACD priorities for the scale-up funding call was to engage with policymakers (Global Alliance for Chronic Diseases, 2019). There was evidence of planned policy engagement strategies, such as including policymakers in the grant applications, involving them during steering meetings and taking their advice for site selection. However, details of policy engagement planning processes varied with some using specific frameworks to guide the process (Ramani-Chander *et al*., 2023).

### Study design

We identified four groups of stakeholders:

1. Principal Investigators (PIs) who had oversight and overall responsibility for the implementation;
2. Other Project Investigators who led particular components of the research such as stakeholder engagement;
3. Project Implementers and staff, who led the work on the ground.
4. Government representatives, who represented federal and regional policy-making in the implementing country.

Four separate semi-structured interview guides targeting each stakeholder group were developed (Supplementary Appendices 3–6). These guides were deductively developed by identifying common elements featured in eight frameworks designed to facilitate the scale-up of complex health interventions including ExpandNet (World Health Organization, 2009; Yamey, 2011; Bradley *et al*., 2012; Chambers *et al*., 2013; Cooley and Linn, 2014; Milat *et al*., 2014; Barker *et al*., 2016; Greenhalgh *et al*., 2017). The broader findings related to scaling up have been reported separately (Ramani-Chander *et al*., 2024). In the current study, we focus on presenting the policy engagement experiences and activities in detail. The specific questions in the interview guides (Supplementary Appendices 3–6) that provided deeper insights into the policy engagement activities have been highlighted in blue text.

### Data collection

Each of the scale-up research consortia included a multidisciplinary team of investigators representing both the high-income countries (HICs) and the implementation countries, the latter primarily being LMICs. Research activities were conducted collaboratively across teams of researchers from all countries. PIs from all 27 funded studies were invited to be interviewed and were asked to facilitate contact with a mix of stakeholders, to be interviewed, from their study. Researcher X completed the recruitment process and conducted all the interviews using Zoom (Zoom Video Communication, Inc., San Francisco, USA). The interviews were audio recorded and transcribed verbatim using a professional transcription service. Researcher Y and Researcher Z were closely involved in all aspects of data collection (McMahon and Winch, 2018).

### Data analysis

The core research team for this study comprised Researchers X, Y and Z who were a part of the working group collaboration but external to any of the funded scale-up projects, thereby providing an independent perspective for conducting this study. We undertook inductive, open coding using thematic analysis (Braun and Clarke, 2006, 2021) to help retain the richness of data obtained, facilitated by NVivo software (QSR International Pty. Ltd, Version 12). Researcher X conducted the coding process, and Researcher Z independently coded 10% of transcripts. The core group worked closely throughout the analytical process (Joshi *et al*., 2009; O’Connor and Joffe, 2020; Keene, 2023). At the end of the coding process, 63 codes generated 15 themes which reflected patterns of meaning shared by the participants. These included both challenges and facilitators faced during the process of policy engagement.

## Results

### Descriptive results

**Figure 1.**
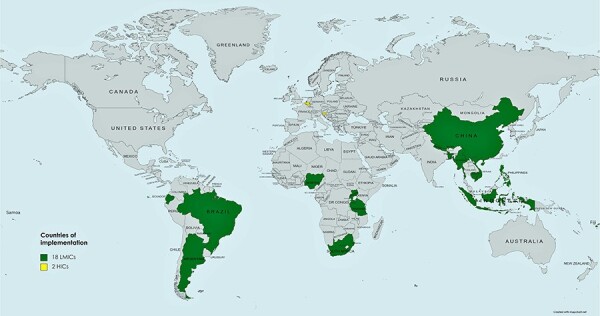
Map showing the global spread of 20 countries of implementation included in this study (*n* = 19 projects)

In total, the PIs from 19 studies targeting populations in 20 countries globally agreed to participate, with 43 semi-structured interviews conducted. The implementing countries were in Asia (eight countries), Africa (five countries), South America (three countries), Europe (two countries) and Oceania (two countries) (Figure 1). Between one and four interviews were conducted for each study, with 12 projects (63%) having at least two team members participating (Table 1).

**Table 1.** Number of participants interviewed from the projects and their role (*n* = 19 projects)

Number of participant/s from each project	Number of projects (*n* = 19)	Total participants (*n* = 43)	Role of participants
1	7	7	5 PIs/Co-PIs 2 Other project investigators
2	4	8	4 PIs/Co-PIs 1 Other project investigator 3 Implementers/staff
3	4	12	5 PIs/Co-PIs 4 Other project investigators 2 Implementers/staff 1 Government representative
4	4	16	7 PIs/Co-PIs 4 Other project investigators 4 implementers/staff 1 Government representative

Sixty per cent of the interviews were conducted with stakeholders located in the implementation country. The remainder (40%) were external to the implementation country, representing research partners located in eight countries across Europe (three), America (two), Asia (two) and Oceania (one). Twenty-four interviews (55.8%) were conducted with females and 19 (44.2%) with males.

Policy engagement during the early implementation phase was characterized by policy dialogues with influential decision makers at both the federal (national) and subnational (district) levels. Within each project, engagement activities were primarily led by the PIs and implementation leads in the implementing countries, with investigators in partnering countries being regularly updated about the progress and any challenges.

Through a detailed thematic analysis on policy engagement, we identified 15 themes and these are summarized with exemplar quotations. We present results themewise and labelled *T1*, *T2* and so forth with numbering cross-referenced between the table and text. Please see Table 2 for details.

#### T1. Ongoing process

One of the most widely discussed challenges in undertaking policy engagement was the significant time and effort required to keep policymakers informed about the study. Discussions with policymakers commenced early, often prior to funding, and were sustained throughout the early implementation phase. Though reported to be tedious in undertaking, this type of ongoing engagement was perceived as being essential for implementation or when seeking any support towards implementation and was a requirement for this scale-up funding call. Due to policymakers’ limited availability, multiple attempts were often necessary to make contact, which was often only brief. This consultative process was time-consuming, yet sometimes yielded only limited clarity regarding commitments. In one case, PIs were considering dropping one country from the implementation plans because of inadequate buy-in and firm commitments from federal policymakers despite early engagement efforts.

#### T2. Its complex

Engaging with different actors in health departments was necessary to understand the varying needs and agendas, and to address them satisfactorily through research. This critical task required skills to navigate the complexities in relationships between policymakers and other actors at different levels. In countries with decentralized governance, separate regional consultation was required to understand the differing needs between the local contexts. Researchers reported added complexities when their engagement had to span multiple government departments as part of their implementation, with each department often requiring consideration of differing interests and perspectives.

#### T3. Role of power

Effective policy engagement also necessitated a good understanding of the internal government and inter-department influence on decision-making towards implementation. In one case, after spending substantial time on the engagement process with implementation partners, researchers discovered that they were collaborating with agencies that had limited policy influence on decision-making or governance of NCDs.

*But after several months we found out, oh you know, perhaps they are really keen. They are still enthusiastic. But they don’t really have the control for the project* (LMIC, PI).

The power and influence of external entities on policy decision-making, though not a widely discussed challenge, presented a formidable barrier to policy engagement for team members in countries, where such behind-the-scenes activities existed. For instance, lobbying by powerful industries who opposed the roll out of alternative products and processes.

**Table 2.** Summary of 15 themes with exemplar quotations

Themes	Exemplar quotations
***T1***. *Ongoing process*: Policy engagement is an ongoing process requiring time and effort.	*Keeping them involved yeah by talking, by phone calls. A lot of people like phone calls. Also, by documentation, emails* (PI, LMIC). *Right now, you know, the national government changed. We started with another one and now we have a new authority at a national level. I don’t know, I guess we spent like 2 months already explaining to them what we are doing. And we are not sure we are going to get the whole network … sometimes you can be tired and say okay forget about it. I quit! But that is part of our life in a developing country* (PI, LMIC). *And what I think is always important is that you have the regular communication as well as the dialogue with the government partners* (Project investigator, LMIC).
***T2***. *It’s complex*: Policy engagement is complex as it requires engaging with different tiers of government health departments with differing needs and agendas.	*At national level we need to show that these national health policies for hypertension and the strategy and the program can be implemented at local level. One of the things the National Health Ministry are trying to develop is for the local level. Then we have to go through the provincial level … the local level, they need time, benefit and results* (PI, LMIC).
***T3***. *Role of power*: Challenges due to power structures that influence decision-making towards implementation.	*But after several months we found out oh you know perhaps they are really keen. They are still enthusiastic. But they don’t really have the control for the project* (PI, LMIC). *[The Food Corporations] are stronger than the government … they are doing a lot of influence on the government all the time … If we have the rules or the norms, they are always trying to stop the government using it and applying it* (PI, LMIC).
***T4***. *Ongoing disruptions*: Maintaining continuity of engagement despite disruptions: Political disruptions, Civil unrest COVID-19 High turnover of government representatives makes it difficult to maintain continuity of engagement.	*The project was supposed to involve [Name of country]. But we found that it is very hard to involve them … the issue is that because they had the big Measles outbreak, so because of the limited resources they have, it has to be mobilised towards Measles. And then after the Measles then came the COVID. It is a small country so you can imagine the limited resources … that is contributing to them not really coming on the table for this study* (PI, LMIC). *We are actually a bit stuck because [Name of country] culture is based around face-to-face interaction. And we had planned that because we were going to have a scale up [Scientific meeting] meeting actually and we had invited all of the region’s health officers and the leaders and government officials to one place. But we can’t do that now because of COVID. So, we are a little bit stuck now. We are basically trying to do it like over Zoom* (PI, HIC). *The difficulties that we always face in [Name of LMIC] and in developing countries is that you can be exposed to many changes, too many health authorities, too many contingency situations. And their plans can change. And then you cannot avoid that but you can be prepared in some way* (PI, LMIC).
***T5***. *Balancing research rigour with practical implementation needs*: Addressing tensions around the practical implementation needs of policymakers with the stringent protocols necessary for producing good quality evidence.	*So, we had to discuss with the Ministries about the program on how you know this [changed decision] affects the project and what’s the way forward. … We have to work together. Because this is an implementation study. So, we want to sort of evaluate what the Ministry is doing. So, we cannot continue with our study the way it was designed if the Ministry is changing the way it is doing its [originally planned process]* (Implementor, LMIC).
***T6***. *Uncertainties on outcomes*: Limited clarity on long-term impact: whether the evidence will lead to policy change beyond the funded period.	*It will work …. The test results need to be good. Even then there is no guarantee that it will be sustained* (PI, HIC). *But if that skill is not transferred to the policy makers or those that would actually be implementing, then chances of sustainability maybe dicey … Because most of the projects are being implemented by [researcher team] and we get to receive the findings of the implementation. And so, if you are not part of the implementation process, then it is going to be very difficult to sustain the gains of the projects* (Government representative, LMIC).
***T7***. *Promote local leadership*: Visibility and leadership of in-country researchers in leading the research.	*… the policy maker was always a challenge to bring them on board. And that is done largely through our [Name of country] research partners … policymakers they are much better to engage if … that is facilitated by [in-country] partners and [the policy makers] see that this is as much an [Name of implementing country] project as it is a [Name of HIC research partner] project* (PI, HIC). *[Name of intervention] is new for them … the proposal sometimes they feel that it is a program from abroad, from United States, from Australia …. They say it seems that it is from a capitalist country* (PI, LMIC). *Because if you are in Europe you think that something will work in [Name of country]. But because you know this is a low and middle income country it may not work. We are the ones who understand how the health system in [Name of country] works and the changes and the challenges we face as we implement whether it is us implementing or … we do it as the Ministry of Health intervention* (Implementor, LMIC). *Useful about the leadership that we have at the moment is we call it in the equal and openness that we are getting. It is not like the partners we used to have way back where the decisions are being made from the [Name of HIC] and you just implement the decisions that has been made in [Name of HIC]. At the moment the decision is equal. It is – the people discuss and decide okay this is the way we go. And they are very happy with this kind of leadership. Really. It brings morale to the work and also you feel like you are fully a 100% part of this program. It was really quite different from the previous partnership that we had* (PI, HIC).
***T8***. *Clarity on roles and responsibilities*: Engagement activities must include clear explanations about the role, contribution and expectations from the policymakers and researchers in the research process.	*We provide clear information to build [policymakers] understanding of what is required. I think when that is missing, that always raises some doubts, and that is when the trust gets shadowed a bit* (PI, LMIC). *You need to bring awareness several times, that they know what you want. Because towards the end of the day we are all working together. One goal. Helping people to improve their health* (PI, LMIC). *But overall we also know that in terms of policies [the data] are going to be very useful … we told them about the general study and by the end it is important for us to have policy document and for these documents to be enforced* (PI, LMIC). *When we, [researchers and policymakers] sit together we say okay, this is what we want to do. How do we go about this? Do you have like an opinion? What is this? What are the gaps?* (PI, LMIC). *Yeah, during, during the phases, they were very interested about our work. And they made suggestions in order to improve our intervention based on their experience in order to serve the country. So we received many suggestions. And we respect them …* (Implementor, LMIC). *That is the ultimate objective, and the research has to be – there has to be complete buy-in from government that the research is of value. The knowledge that will be generated is of value to them. Otherwise this type of research doesn’t make sense* (PI, HIC).
***T9***. *Work within government structures*: Policy engagement requires an astute understanding of the local government structures, bureaucratic processes and decision makers.	*Apart from the [Name of province] I didn’t know any of them in the other provinces. So that was why the introduction and the support of the national department was quite crucial you know. Because there is this sort of hierarchical system you know. People are more likely to give you some attention if the National Department is promoting the idea. You know if you just come without anything then you are just another voice crying in the wilderness and they may or may not take any notice* (PI, LMIC). *When we started the [name of previous project] … we had written a letter introducing the projects right from the government through to the [Title of highest ranking health policymaker] for the state. And then trickled down to other heads, some agencies, relevant agencies that are required for that project* (Government representative). *You need to know that there was genuine support … from the sub-district and district level. And somebody was going to monitor whether this happened or not* (PI, LMIC).
***T10***. *Invest in building trust*: Requires trust and strong relationships: Established through previous research. Must be strengthened through engagement.	*Yeah I think definitely relationships, trust and histories are important* (PI, LMIC). *I think [past experience] builds trust, it builds rapport, particularly with [Name of Implementing Country]. I’d worked with the [name of health] department there. I mean I have interacted with [policymakers] once or twice a year for 15–20 years. I think a lot of this is about trust that they don’t feel that you are going to come and use them for your own benefit. I think with time they see that you do give something back and then that helps. Those links … which are built up over time. Trust is absolutely critical in all of this* (PI, HIC). *So [PI, LMIC] has that technical capacity more or less to implement the experience …. Two, he also has a good stakeholder management, himself and his team. They understand the landscape of [Name of implementing country] and they know how to manage stakeholders in achieving a project’s objectives and goals. They have been able to successfully do that in the past project and in the GACD project. So they are very good at managing stakeholders and ensuring—discussing different levels of stakeholders and ensuring that we achieve, we all achieve the aims* (Government representative, LMIC). *We are really close. We make some friendship links and we are really close. And the decision is all the group making together* (Implementation lead, LMIC). *If we want them to work together, we have to build this relation and address the relation. And you don’t achieve this in one day, in 10 days. You have to facilitate and create this relation … this is not so simple. Because we are talking about people … you have to make people trust each other* (PI, LMIC). *It is very important because we are coming from an institution, university, and our project investigator especially linking up with the Ministry of Health. Because they do not want our project to be seen as ‘ours’. So in terms of ownership, we are trying to do things to help, be in line with the priorities of Ministry of Health of a government. That is why it is important to always link with them, and make sure that they are actively engaged in the project* (PI, LMIC).
***T11***. *Explain and educate about implementation research*: During engagement researchers must clearly explain the purpose of research and how it will help provide evidence for making policy decisions: Local evidence. Role of pilots.	*The global practice, global evidence is not enough for [Name of country] government to take final decisions about the interventional approach … they support their scientists and research entities to collect local data and evidence and then consolidate for the policies* (Implementor, LMIC). *We manage with the situation in our pilot by having personal contact with [policymaker]. And explaining them how damaging this situation can be for the health of the communities. And showing them numbers …. So we try to explain them how this can be bad for their politics for the view of the communities, of their politics … we have meetings and explaining the project and the value and importance of the project to minimise these difficulties. And it worked!* (Project Investigator LMIC). *We piloted it in north regions, and it proves that it is feasible and this can be managed by primary level facilities by our nurses. So that informed that this can then scaled up* (Government representative).
***T12***. *Frame research benefits within local priorities*: Describing the outcomes of the study within the larger policy and health agendas of different tiers of government: evidence of population health benefits. evidence of community buy-in.	*Of course, one of the key things when we went to the provinces as well as the National Department was that we are really carefully articulating how this initiative dovetails with the policies they are currently sort of engaged with …. So … in our presentation we had to say this is how it fits into the [xxx] policy. … [xxx] Policy, and the [xxx] Policy … So you have to show very clearly how this thing fits in to the policy direction and the things that they want to kind of implement. And how it is going to help them implement their policy. So that was a very critical piece … because they are actually quite policy driven obviously and they want to deliver the main policy* (PI, LMIC). *The fit that [the community] got. The really appreciated that the services finally will be brought closer to them. Because … they would leap over the tertiary level for them to be in a position to see the doctor who so ever will provide services at the tertiary level facility. And they would need to sometimes bear the impact, to pay a visit they need to go as a group so that they can take it to the facility. Yet now it is good news for most of them* (Government representatives, LMIC).
***T14***. *Unify NCD action through research*: Fostering cohesive action within the implementation country: through vision alignment. creating advocates for NCD research. joint workshops.	*It is buy-in from the individual, from the personal linkages that those city health offices have with some of our core [Implementing country] staff is very important. It is not just that. It is something also around whether they personally think it is going to help the people in the villages that they are enlisting the help of* (PI, HIC). *Because to gain sustainability, it is … my opinion that the projects like these are moved by persons. But not one person. A team … you have to create relations …* (PI, LMIC). *As always you need leadership. You don’t have a good project if you don’t have a good leadership … [Implementation lead] is a very good leader, the [Position of leader] is a good one. The President of this Administration of [Name of partnering institution] is a very good one. There are a lot of very good leaderships so it is you know like a dream team* (PI, LMIC). *So there are some kind of liaison person which uniting as a system … But all of us luckily enough looking at the same orientation, same direction* (PI, HIC). *We can assume that we are sort of moving in the same direction and have the same goals and visions for what difference our research might be able to make. How practical we want to be and that kind of stuff. So it’s an aligning of purpose and approaches* (Project investigator, HIC). *Sometimes we are in our main job is doing research. Some other times our main job is building bridges between people in government and people in the [name of movement]. And sometimes we are also training people in the [name of movement] based on these new evidence that we are building and we have been building for long time starting in all those projects 30 years ago* (PI, LMIC).
***T15***. *Be inclusive about dissemination*: Planning for comprehensive dissemination	*You know obviously it is how you disseminate back, but it is really done in the sense of how do we help you to be more effective as opposed to you know, we are judging you* (Project investigator, HIC). *So it’s really not sort of saying this didn’t work, but … here’s what we found and here’s the potential challenges. Help us understand you know are there potential opportunities for how this could be implemented better etc. it is sort of how you share and don’t give people surprises and certainly don’t ever tell them something in public that has not already been discussed!* (Project Investigator, HIC). *We are asking city health offices if they think the information we are providing is appropriate? Do they want it provided in different levels, different ways? Do they want it visually, do they want videos? Definitely the team have maintained close connection with them* (PI, HIC).

#### T4. Ongoing disruptions

Disruptions to policy engagement due to political upheavals and civil unrest were commonly faced across projects. At times, this resulted in changed government priorities, or reduced support, potentially impacting implementation in a significant manner, as explained by the following quote.

*They want these models. But governments can change, right, and might find that there is some element that doesn’t work … and then government might say, well, without those elements I am not ready to do it* (PI, HIC).

COVID-19 was a common challenge, with policymakers from the Ministries of Health redirected towards managing the pandemic response. As the pandemic emerged, all policy engagement activities planned by the research team stalled, with little, or no, clarity on availability or progress. However, some team members, interviewed later in this study, reported commencing or resuming discussions with policymakers. The rapid acceptance of online meetings and platforms such as Zoom or WhatsApp facilitated continued engagement. However, the quality of interactions and relationship-building offered by online settings may have compromised engagement, with influential policymakers across LMICs generally favouring personal over online meetings.

High turnover of government representatives was a formidable challenge for engagement. Researchers and implementors shared their frustrations at the wasted effort when policymakers, with whom they had engaged with extensively, abruptly changed following government elections or other reasons such as retirement or better employment opportunities. As explained by the following quote, this often required recommencing consultation and negotiations with a new set of policymakers, potentially compromising the outcomes.

*… like the staff of health government sometimes also changes … because it is new person – they are always asking what is [name of intervention]* (PI, LMIC).

One way in which the team members navigated this challenge was by requesting the retiring policymaker to introduce the research study and team members to the new policymakers, thereby gaining some potential for continued support.

#### T5. Balancing research rigour with practical implementation

PIs shared significant tensions around balancing the rigour of research protocols with the practical needs of policymakers. Implementation of stringent research protocols was often not prioritized within the government departments, or protocols changed, creating considerable challenges to researchers. Conversely, policymakers explained that sometimes their policy decisions required immediate evidence, such as in their response to COVID-19. Further, one policymaker highlighted that, because of their limited budget and competing health priorities, they often could not prioritize or allocate sufficient resources towards the implementation efforts. These divergent viewpoints precipitated negotiations around the need to maintain rigorous standards and procedures for providing reliable scientific and actionable evidence. This often resulted in internal escalation within the research partnership to include international investigators in these negotiations and sometimes leading to modifications in the study design.

#### T6. Uncertainties on outcomes

Despite investing time and effort in the consultation process, researchers expressed concerns about the usefulness and long-term impact arising from engagement efforts, and whether the research would truly result in policy change. From their view, policymakers were sometimes enthusiastic and willing to engage but far more conservative when it came to making actual commitments. One policymaker stressed the need for researchers to involve government staff adequately during the implementation stages of research so that local staff would develop the necessary skills to sustain the changes.

#### T7. Promote local leadership

Policy engagement was mainly undertaken by PIs in the implementing country supported by research team partners and members. This process was generally perceived to facilitate policy engagement as it appeared that some policymakers trusted in-country researchers more than those from elsewhere. The active involvement of in-country researchers inspired trust that local knowledge and realities underpinned the research. Promoting and encouraging leadership from the researchers in the implementing country were considered fundamental to building an equitable partnership, within the research consortia. Establishing this culture early in the research process helped to emphasize the role of contextual knowledge in implementation science, promoted reciprocal learning and local decision-making, which together supported local leadership.

#### T8. Clarity about roles and responsibilities

Researchers reported benefits in spending time explaining implementation research and in clarifying the roles of researchers and policymakers during the early engagement phase. Because implementation research is an emerging field, particular engagement efforts were directed towards explaining how the research could support effective scale-up of interventions, an important need for governments. Some PIs also shared the importance of clarifying the nature of support required from policymakers both during the research and on an ongoing basis to effectively support the research implementation. As elucidated by the following quote, this transparency set expectations and enhanced commitments while also building trust.

*So, we provide clear information to build their understanding of what is required of [policymakers]. I think when that it is missing, that always raises some doubts, and that is when the trust gets shadowed a bit* (PI, LMIC).

Encouraging policymakers to contribute to the research agenda acknowledged their expertise and respected their vital role in the implementation process. Further, enabling policymakers to shape the research agenda was perceived to foster a sense of ownership among them and garner some support for the research. These strategies also helped to ensure that the research output was meaningful to the policymakers, thereby securing long-term commitment to the research and the utilization of evidence in guiding policy agendas.

#### T9. Working within government structures

Effective engagement with policymakers necessitated an astute understanding of the local governance structures and power dynamics. This understanding helped PIs to identify the influential decision makers within the government with whom they could engage during implementation. Across studies, team members engaged at the federal level first, securing firm commitments often in the form of letters of support, or personal introductions which helped later engagement with subnational policymakers. Such documentation also marked important milestones that demonstrated the firming of federal commitments. Policymakers also shared the importance of drawing up strategic plans for scaling up, such as the selection of regions or sites, based on the local conditions and prevailing politics.

#### T10. Invest in building trust

The presence of trusting relationships between the team members and influential policymakers, developed through experience and context-driven research, provided pathways to facilitate engagement, while the engagement further strengthened relationships. In this context, securing individual policy-level buy-in was viewed as a critical step towards securing broader institutional buy-in.

Researchers shared the view that trust on four fronts helped them engage with policymakers, (1) by expressing genuine intentions of the researchers to improve the health of the local population, (2) on the expertise and skills possessed by the researchers to deliver what the policymakers needed and what was best for the country, (3) on the personal attributes of the researchers, such as respect for the local culture and context and (4) by clearly establishing local country ownership of the research, i.e. led by local researchers, for the benefit of the local population, and providing evidence to support local policy-making.

Relationship strengthening was critically important during early implementation. Notably, COVID-19 activities provided the opportunity to strengthen relationships, whereby researchers were able to provide timely help to policymakers, e.g. by offering epidemiological assistance or supporting other aspects of COVID-19-related research as per the policymakers’ requirements. Policymakers also expressed appreciation for in-country PIs for their enduring commitments to population health and research, and on the ability to manage and motivate diverse groups of stakeholders. Strengthening these existing policymaker–researcher connections was an important part of relationship-building activities undertaken during the engagement process.

#### T11. Explain and educate about implementation research

Researchers reported that policymakers, in general, were supportive of research studies that focused on procuring local evidence to address local needs and conducted by researchers with contextual knowledge. Therefore, focusing conversations around establishing evidence on the local context through co-production with the policymakers facilitated policy engagement. This enabled researchers to explain how they could contribute meaningfully to assist governments, while providing opportunities to understand the policymakers’ viewpoints, needs and limitations.

*We aim to develop those in co-creation with the stakeholders. So mainly through policy dialogues* (PI, HIC).

Having prior feasibility or pilot studies was useful for policy engagement as it allowed the researchers to showcase the benefits of developing contextually relevant insights. Such studies were also pivotal in providing an avenue for undertaking large-scale implementation, as they had already provided a solid foundation for how implementation works and in establishing trustworthy relationships that could potentially favour the scale-up process. Researchers also shared how providing local data and other resources to strengthen civil society organizations, and building population awareness on behavioural risk factors through education, indirectly, but effectively, exerted influence on policymakers to engage and act on NCDs.

#### T12. Frame research benefits within local priorities

Researchers expressed the importance of tailoring their policy dialogues to address the varying needs and requirements of different tiers of government. Through policy engagement, researchers aimed to directly address how they could collaborate with policymakers and provide evidence that was in line with local government policies and priorities. These dialogues enabled them to clearly position the implementation research as a means to support policy decision-making for improving population NCD health outcomes, improve well-being of the community and improve delivery by frontline staff. Framing these conversations in this way empowered the policymakers to consider their government needs and requirements from research, facilitating a more collaborative process of co-production.

#### T13. Plan communication strategies

Researchers across studies reported how benefits arose from keeping policymakers regularly updated about the progress of the study, including sharing important research milestones. Regular communication helped elevate the research study’s visibility at the policy level, and facilitated the ability to discuss and understand mutual needs, maintain policy involvement and support local decision-making and ownership. Importantly, while seeking continued cooperation from policymakers, researchers were also able to convey respect for the important role of policymakers in the research process. As illustrated by the following quote, such communication led to the development of equitable partnerships:

*[Name of LMIC PI] and his team is very good at the communication and making sure that the findings are owned by the people who are the decision-makers as opposed to a feeling that we are doing something to them or about them … that we are doing with them* (Project investigator, HIC).

Team members reported that prior to formal engagement, policymakers appeared to require some level of trust and rapport with members of the study team, particularly the lead investigators. These easy-flowing conversations provided a sense of camaraderie that was an important facilitator to engagement efforts. This was achieved by adopting a balanced approach that combined culturally appropriate, relaxed and informal communication together with more formal presentations.

#### T14. Unify NCD action through research

Team members shared how different government departments in LMICs often worked in a fragmented manner, and therefore a significant part of their policy engagement activities was directed towards building communication and developing a common shared vision of improving population NCD health. Organizing both individual- and group-based engagement provided the optimal balance between understanding individual department or policymakers’ needs and developing wider policy partnerships.

We identified that team members were not only engaging to gain the support of individual influential policymakers or actors for the duration of the study, but that the ultimate goal was to also build a team of advocates or a ‘coalition of champions’ (Damschroder *et al*., 2009). This was important as these decision makers would help to continue to prioritize the scale-up of NCDs, within their respective spheres of influence, and even beyond the scope of the current funded study. We show that the identified policy champions were often local leaders who understood the gravity of the NCD burden in their communities, were passionate about improving local community health, and generally accepted the role of research in this process. Researchers often intuitively identified these leaders and engaged with them in a more strategic manner.

#### T15. Be inclusive during dissemination

Dissemination of research with high-level policymakers was an important part of policy engagement to share early findings of results. Researchers found benefits in explaining the short- and long-term implications of the research rather than sharing the research findings alone. Using simple language supported by visual charts supported communication and aided understanding of scientific results. Some researchers described how consulting with policymakers to identify how they preferred receiving the dissemination materials helped to improve the relevance and usefulness of evidence provided. In the longer-term, policy-level dissemination sessions were considered particularly effective when conducted as joint workshops including with the wider stakeholder networks. Examples were provided on including, or plans to include, different government leaders and decision makers, community members and implementors under the same roof to provide a forum for joint reflection of results. Additionally, establishing project-related governance mechanisms, such as steering committees that provided a forum for facilitating discussions and steering study goals, were important to improve the policy–research interface. These committees involved policy leaders and promoted the dissemination and sustainability of research findings into policy decision-making.

As identified by the thematic analysis, several strategies were adopted to support and encourage policy engagement, and these have been summarized in Box 1. These strategies are a summation of the actions, processes or factors that researchers considered as facilitating policy engagement with a view to sustainment at the policy level. While some of these strategies may have been experienced, others were anticipated benefits arising from the engagement being undertaken during the early implementation phase.

Box 1. Summary of recommendations that facilitate policy engagement during the early implementation stage

Understanding the policy landscape

- Identify influential policy-level decision makers and government-based champions.
- Having a strategic understanding of the influence of individual policymakers and power dynamics.
- Past research contribution and experience in the implementation country is helpful.
- Previous feasibility or pilot studies provides contextual knowledge and connections.
- Having trustworthy relationships between researchers and policymakers.
- Developing transition and contingency plans when policymakers change.

Creating an environment that supports co-creation

- Engagement with policymakers led by in-country researchers.
- Framing conversations around the importance of local evidence to gain support from policymakers.
- Policymakers contribute and guide research decisions such as selection of interventions, or study sites.
- Using clear, transparent and non-judgemental language during communication.
- Sharing bite-size, digestible bits of information and findings to encourage scientific understanding.

Fostering a culture of two-way learning

- Being clear about the roles and expectations of policymakers and researchers, before, during and after the study.
- Using the national NCD policy in research.
- Explaining research output in simple language and relating findings to population health benefits.
- Understanding the practical needs of policymakers and discussing how researchers can assist in that effort.
- Empowering local leadership.
- Being adaptable to provide clarifications or information when necessary.
- Continuous engagement is required to keep the study prioritized at the policy level.

Engagement beyond research

- Fostering partnerships, aligning vision and creating NCD advocates.
- Organizing joint workshops during dissemination including different government departments and wider stakeholder groups including community members and frontline.
- Supporting policymakers outside research programmes (e.g. engagement during the pandemic).

## Discussion

We identified 15 themes when undertaking policy engagement for implementation studies supporting the scale-up of NCD interventions. These themes reflected a combination of both challenges or facilitators to the policy engagement process. Strategies to engage with government stakeholders leveraged on the strengths of previous work experiences and relationships, while forging new, stronger partnerships was central to the engagement process. Effective communication maintained throughout the early implementation phase supported policy dialogue, co-production of research and promoted equitable collaborations. Focusing on local government policy needs and the stakeholders needs facilitated engagement and empowered policymakers to consider their requirements from research. Non-judgemental, transparent communication using simple language was critical to improve the understanding of implementation research and to develop a shared understanding of the needs of policymakers and researchers.

From this analysis, four cross-cutting concepts were identified, comprising the importance of understanding the policy landscape; the identification of suitable policy champions and creating a network of champions; the need for equity to empower local policy leadership; and the creation of a unified policymaker–researcher lens towards supporting implementation research.

### Understanding the policy landscape

Implementation science promotes the uptake of evidence-based interventions into policy and practice, and engagement with policymakers is critical in this process (Peters *et al*., 2013; Global Alliance for Chronic Diseases, 2019; Skivington *et al*., 2021; Marten *et al*., 2021). Governments and policymakers influence priority setting, facilitate wider use of the intervention and influence decision-making (World Health Organization, 2010; Peer and Kengne, 2016; Gore and Parker, 2019). In addition, co-production of research, which necessitates researchers and policymakers working collaboratively, promotes long-term sustainability and policy impact (Haynes *et al*., 2020; Erismann *et al*., 2021). However, co-creation and co-production require time and engagement with the right stakeholders and efforts to consider their priorities, a lesson that is supported by the findings in this study (Lazo-Porras *et al*., 2020; Beran *et al*., 2021). We also highlight the critical importance of understanding the local landscape, identifying relevant decision makers and navigating the prevalent bureaucratic processes when undertaking policy engagement for scaling up.

Policy analysis involves understanding the processes of policy-making, the actors involved and the context within which they operate (Walt and Gilson, 1994). It employs various methods and tools to explain the policy establishment (Collins, 2005), and the prioritization of policies at both the institutional and individual actor levels (Gilson *et al*., 2008; Buse *et al*., 2009). Policy analysis is also a useful tool to identify stakeholders who may support or resist reform and thereby help decide how much effort and resources are required to build that relationship (Mugo *et al*., 2020).

In the realm of implementation studies, engagement plans for policymakers are often considered as a part of the stakeholder analysis, and there are several frameworks and tools available to conceptualize and facilitate this process (Young *et al*., 2014; Goodman and Sanders Thompson, 2017; VicHealth, 2019; Bernstein *et al*., 2020; Eisman *et al*., 2021; Grill, 2021; NHS England and NHS Improvement). We provide evidence that prioritizing and conducting policy analysis during the formative stages of planning, can potentially enhance the efficiency and effectiveness of the policy engagement process.

### Empowering local policy leadership and creating a ‘team of policy champions’

The scale-up process requires policy actors to drive the NCD agenda forward. In a recent study on stakeholder engagement for scaling up digital health interventions, the authors explained the importance of the role of ‘future champions’ who facilitated change within their organizational units (Lampariello and Ancellin-Panzani, 2021). Our findings highlight the need to engage with local influential government-level decision makers and facilitate partnerships between these different champions to realize the coherence of policy and vision of change across sectors and tiers of health policy-making. This necessitates the formation of a coalition of like-minded actors and advocates who possess the leadership skills to prioritize NCDs within their respective roles and capacities (Hunter *et al*., 2019; Lampariello and Ancellin-Panzani, 2021).

While we found that researchers often used their intuition to help identify the local champions, using a matrix such as the 3D grid of power (ability to influence), interest (extent of activity or passivity) and attitude (the extent of support) to map policymakers may better identify a suitable network of government stakeholders (Murray-Webster and Simon, 2006; Bernstein *et al*., 2020).

### Providing an environment for co-production and local leadership

The third high-level meeting of the General Assembly on NCDs in 2018 discussed the importance of strategic leadership from heads of state to improve the action towards NCDs (World Health Organization, 2018). This need for greater policy coherence was further discussed in the implementation road map document of 2023–30 (World Health Organization, 2021). Local leadership supports local ownership and provides the responsiveness required for dealing with rapidly changing situations (Theobald *et al*., 2018), and was one of the identified reasons that promoted the success of HIV programmes (Rabkin and El-Sadr, 2011). We highlight that local leadership also promoted trust in the research process, leading to improved policymakers’ engagement. Similarly, developing connection with influential policymakers and leaders was also a facilitator. This raises the need for a deeper discussion on addressing power dynamics within two important partnerships to foster local leadership during policy engagement: (1) between the research collaborators and (2) between researchers and policymakers.

International collaborations play a crucial role in providing the breadth of disciplinary skills necessary to undertake comprehensive scale-up research projects. However, if research expertise and leadership is a facilitator to policy engagement, then it is essential that the governance and planning within such partnerships prioritize principles of equity, integrity, leadership and reciprocal learning (Binagwaho *et al*., 2013). Careful consideration of the roles and responsibilities within the research consortia will promote legitimate contribution from the implementation country team to a wide range of collaborative efforts, thereby enhancing research capacity and leadership (Godoy-Ruiz *et al*., 2016; Parker *et al*., 2016; Engelgau *et al*., 2018; Faure *et al*., 2021; Murphy, 2022).

Similarly, the role of collaboration between researchers and non-researchers, including policymakers, is essential for co-production, local contextualization and for lasting policy and practice impact (Ghaffar *et al*., 2008). But this will only be possible if the purpose, intent and conduct of such research aim to foster more equitable, culturally sensitive and reciprocal relationships (Ezeanolue *et al*., 2018; Oni *et al*., 2019; Beran *et al*., 2021).

### Promoting a two-way learning during engagement

Policymakers and researchers may have different perspectives on the research process. While we found that policymakers were supportive and encouraging of local researchers and gaining evidence on their local context, they may not fully appreciate the processes involved in conducting such research. This could be attributable to many reasons, including insufficient understanding about implementation research in particular, not having adequate experiences or opportunities to have been involved in research that truly benefited them, or purely arising from having a completely different agenda (Uzochukwu *et al*., 2016; Peters *et al*., 2019). On the other hand, researchers through their training may not fully appreciate the practical needs, challenges and limitations of policymakers.

Focusing early discussions around the benefits of implementation research, explaining the role of co-production and having clear conversations around ownership will improve the understanding of research between the two groups (Uzochukwu *et al*., 2016; Collins *et al*., 2022). Similarly, researchers must develop an understanding and appreciation of the needs and limitations of policymakers. These strategies will help unify the perspectives of researchers and policymakers views into one cohesive lens of ‘implementing research to inform local policy decisions for scaling up of NCDs’, and potentially promote the uptake of research into policy decision-making. Further research is needed to capture the perspectives of policymakers towards research when scaling up interventions targeting NCDs. This is critical to understand insights into the dichotomies that commonly distance policymakers and researchers.

## Strengths and limitations

There are several strengths to this study. Firstly, the sample size of 43 interviews with team members from 19 globally implemented programmes offered rich and varied insights into the policy engagement processes. Secondly, this study was conducted during the early implementation phase allowing us to capture the experiences as they were experienced. Thirdly, documenting the impact of COVID-19 on policy engagement, a common challenge faced across all these studies, adds valuable context.

Further, while our findings focus on policy engagement to support the scaling up of NCD interventions, it is equally valid and helpful to all implementation researchers working on NCDs. Pure implementation is the process to integrate interventions into practice while scaling up (using implementation science) is expanding evidence-based interventions proven to be effective in controlled conditions to real-world settings (World Health Organization, 2009; McKay *et al*., 2019). Scaling up is therefore more complex and requires broader consideration of policy engagement processes to encourage participation and support at multiple levels to shape national policy agendas and regional agendas on issues such as prioritizing of NCDs and funding (Ajisegiri *et al*., 2021; Collins *et al*., 2022). Implementation science provides the systematic evidence to improve the quality and effectiveness of interventions and promote uptake into policy and practice (Eccles and Mittman, 2006), and can be embedded during both small-scale implementation and larger scaling up efforts (Marten *et al*., 2021; Collins *et al*., 2022).

However, it is important to acknowledge certain limitations. Firstly, we would like to acknowledge that while we are providing detailed practical insights into the policy engagement activities during the early implementation phase, the interview guides were developed through an analysis of scale-up frameworks and not policy engagement frameworks, potentially limiting the overall exploration of relevant issues from policy literature. Secondly, we were not able to interview research team members from all countries of implementation. Similarly, policymakers in all countries were extremely busy during the pandemic and we were able to interview only a small sample. This limited our capacity to comprehensively understand the stakeholder perspectives across all studies, potentially limiting the generalizability of these findings. In doing so, we recognize that we may be presenting dominant stakeholder views and largely from a non-policymaker lens.

Thirdly, the interviews were undertaken over 10 months, in a phased manner across the studies coinciding with the pandemic. Since the global situation evolved rapidly, the experiences of team members towards policy engagement likely varied from the beginning to the end of the study period. It is worth noting that the influence of COVID-19 may have overshadowed other challenges that may have arisen during normal implementation efforts. Moreover, since this study was focused on capturing stakeholder experiences on policy engagement during the early implementation phase only, we are unable to reflect on the overall impact of COVID-19 and any adaptation to the projects as a result of this. Furthermore, we report the collective experiences on policy engagement across all the funded studies, without divulging project-specific details on the policy engagement activities undertaken within each study.

## Conclusions

This study, conducted between 2020 and 2021, coincided with approximately one-third of the time frame set for achieving the sustainable development goals (2015–30), which included targets for reducing the global burden due to NCDs. While several hurdles lie in the path of gaining sustained commitment from policymakers, it is encouraging that several strategies can be employed by research teams to address these challenges. We recommend that researchers conduct formative policy analyses to support an efficient engagement process. Power inequalities must also be addressed by building a culture of equity and respect, so that team members and policymakers can genuinely contribute to co-production. Finally, breaking down research and policy division, through improved communication and education, will lead to unified action and enhance the uptake of implementation research within policy.

## Supplementary Material

czae043_Supp

## Data Availability

The data underlying this article cannot be shared publicly due to the sensitive nature of this research and to protect the privacy of individuals and studies that participated.

## Reflexivity statement

The GACD research network includes researchers, academics and implementors from over 73 countries globally. This study has been conducted as a part of the research activities of the GACD Upscaling Working Group and forms a part of the published protocol, which was co-designed and co-authored by members of this group. The working group membership is open to all project members from any project funded under the GACD umbrella, who are interested in developing the science of scale-up irrespective of location. Every participating study that was examined as a part of this research represents a research consortium with research partners spread across both HICs and LMICs. Project lead investigators from both the LMIC and HIC were invited to participate in this study. Participation was voluntary, and we sought consent from the lead investigators from these consortia. The co-authors for this paper include writing group members from both HICs and LMICs.
