# Supplementary material for: Strengthening policy engagement when scaling up interventions targeting non-communicable diseases: insights from a qualitative study across 20 countries
Source: Health Policy Plan. 2024 Nov 18;39(Suppl 2):i39–53. doi: 10.1093/heapol/czae043 (PMC11570794; doi:10.1093/heapol/czae043)
Supplement: czae043_Supp [file czae043_supp.zip › suppl_data/Appendices_3_to_6_Interview_guides_blinded_ver2.docx]

**Appendix 3: Timepoint 0 (T_0_)- Baseline Interview guide for Principal Investigators (or representative identified by them)**

**___________________________________________________________________**

Thank you for agreeing to take part in this interview. My name is X and I am undertaking this interview as a part of my PhD Research the title of which is “Applying a systems lens to identify challenges, enablers and barriers to the GACD scale up interventions”. My supervisors for the PhD research are Prof. Y from University 1 and A/Prof Z from University 2.

The aim of the interview is to collect baseline information about your scale up study

1. *Can you tell me a little bit about yourself and your motivations that led to this scale-up project*
2. *Please tell me a little about the scale-up project?*
   1. Can you explain briefly what is being scaled-up?
   2. What was the opportunity to scale up? Why?
   3. Is there anything new or novel about what is being scaled-up?
      1. If it is new, what is new about it (e.g. a technical innovation/process (new delivery approach), organizational innovation (new partnerships), or a combination)?
   4. What was the architecture for this scaling opportunity?
   5. Who were the actors and players?
   6. As a PI do you think the scale-up is a fairly simple model or is it complex? Why?
   7. Is the scale-up a horizontal or vertical scale-up?
   8. Is the scale up model selected based on what actually will happen if government/health system will take over? If not, how might it change?
   9. Are there any risks, fears or weaknesses?
3. *Please tell me a little bit about the partnering organisation?*
4. What is its role?
5. In your opinion what are the main strengths that the organisation brings?
6. What are the challenges that you think the organisation will face during the scale-up process?
7. *Could you tell me a little bit about the Chief Investigator/s?*
8. What expertise do they bring?
9. How well do you know them? Have you worked with them previously?
10. Does it make a difference if you know them well or not? Why?
11. How do you, the Chief Investigators & other key partnering organisation members plan to keep in touch during the process?
12. *Who you think are the relevant stakeholders and end-beneficiaries in your project?*
    1. Have you started consulting with them?
       1. If so, how?
       2. If not, at what stage will you be consulting with them?
    2. What is their role? What are the challenges in ensuring their buy-in?
13. *Could you tell me a little bit about the governance structure of the project team and collaborators?*
14. How is the project structured to enable input from all stakeholders?
15. How do you meet (e.g. Zoom, face-to-face) and how often?
16. How are decisions made and who makes the final decision regarding project processes?
17. *What do you think about the health system in the countries where the project is being implemented? Alternative questions for those who have projects that do not involve the health system: What do you think about the health system and other relevant system in the countries where the project is being implemented?”*
    1. Is it generally open to change and receptive and encouraging to new ideas?
    2. What are the critical challenges that the health system poses with regard to implementation/adoption/scale up of the project? How do you know this?
    3. How does the project design take into account the health system challenges? If you project is across several LMIC countries how does the design reflect the changing local system?
    4. We noted in the proposal that you have included process and economic evaluations. Could you comment how you think the findings from these evaluations may address the health system challenges and inform the end-users?
18. *Are there any industry stakeholders for this project (specific probe if not mentioned in 7 above, or for expansion)?*
    1. Who are they?
    2. What is their role? What are the challenges in ensuring their buy-in?
    3. At what stage will you be consulting with them?
19. *Please tell me a little about the local government and key government stakeholders (specific probe for expansion).*
    1. What is their role and how important is their role?
    2. What do you think are the challenges in partnering with them?
    3. How do you plan to ensure their buy-in and what stage of the project is this important?
    4. Do you see any ongoing issues with regard to this?
20. *Are there any other health service providers who are involved in the delivery of the program, in the technical advisory board, designing the intervention, or otherwise involved in this project (specific probe if not mentioned in 7 above, or for expansion)?*
    1. What is their role?
    2. How do you plan encourage them to participate and contribute?
    3. What are the challenges in keeping them motivated and interested?
21. *Tell me a little about the end-beneficiaries of this program*
    1. What are their health needs currently with regard to hypertension and diabetes?
    2. How are these needs being met currently?
    3. At what stage of the program implementation are they being consulted and in what manner?
    4. Why do you think they will adopt and continue with the model/innovation?
    5. Has the same group of beneficiaries successfully taken up any program previously? Is there a history of acceptance?
    6. What do you perceive to be the main challenges in their uptake of the program?
    7. How do you plan to deal with these challenges?
22. *Tell me a little bit about the front-line staff who will be delivering the program?*
    1. How important do you think their role is?
    2. At what stage of the program is their role most important?
    3. Do they have other conflicting projects or programs that they work in?
    4. What are their challenges and motivators to promote this programme?
    5. What is the incentive for them to promote this program?
    6. What sort of resources are being invested in their training? Is there written documentation of all policies that they need to follow?
    7. Has any training material been prepared for their work?
23. *Has a pilot been conducted? If yes:*
    1. When was the pilot conducted? Where was it conducted?
    2. Was it in the same location as the scale up? (same local environment or different?)
    3. Was the pilot evaluated? If so, what documentation and methods were used to gather data?
    4. How did you or the team assess that the pilot program could be scaled up? How do you know that the program is effective (that it works)? How do you know that the program is an efficient way of delivering to the community?
    5. What were some of the challenges that the team faced during pilot stage? How did you deal with them?
    6. What were the main learnings from the pilot? Do you think that the pilot provided any tweaking to the original program strategy?
    7. What were the learnings about stakeholders- motivators, buy-in, challenges/ barriers?
    8. What were the challenges that arose from the health system perspective?
    9. Has the intervention changed because of the results of the pilot?
    10. How are you measuring fidelity?
    11. Did you undertake a cost-effectiveness of the pilot intervention? If yes, how did you use that information for scale up? If not done at pilot, then why not?

*If no pilot was conducted:*

- 1. *Could you please explain why a pilot or feasibility study was not conducted?*
  2. How does the team plan to scope the study, identify local challenges and barriers, and determine what works well and what does not work well?

1. *Please tell me a little bit about the scale-up plan and strategy*
2. Is there a specific plan or strategy that is going to be used to guide the process? Has it been documented?
3. Which members of the team, partners and other stakeholders are aware of this strategy?
4. Which members of the team, partners and other stakeholders have been consulted while drawing this up?
5. Have you used any framework to guide the scale up design and strategy? Why did you apply that particular framework? Did you consider any others?
6. Do you see any potential issues for long term sustainability of the program?
7. What do you think will happen if a new unplanned challenge emerges during implementation? What qualities do you think your team or organisation has to cope with this? How will the team know and respond?
8. Are you measuring cost-effectiveness of scale up? If so could you briefly explain how? If not, why not?

**Appendix 4: Timepoint 0 (T_0_)- Baseline Interview guide for other Investigators and Project Team Members including implementers**

**___________________________________________________________________**

Thank you for agreeing to take part in this interview. My name is X and I am undertaking this interview as a part of my PhD Research the title of which is “Applying a systems lens to identify challenges, enablers and barriers to the GACD scale up interventions”. My supervisors for the PhD research are Prof. Y from University 1 and A/Prof Z from University 2.

The aim of the interview is to collect baseline information about the scale up project

1. *Can you tell me a little bit about yourself and your motivations that led to this scale-up project*
2. *Please tell me a little about the scale-up project?*
3. Can you explain briefly what is being scaled-up?
4. What was the opportunity to scale up? Why?
5. Is there anything new or novel about what is being scaled-up?
   1. If it is new, what is new about it (e.g. a technical innovation/process (new delivery approach), organizational innovation (new partnerships), or a combination)?
6. What was the architecture for this scaling opportunity?
7. Who were the actors and players?
8. As a CI do you think the scale-up is a fairly simple model or is it complex? Why?
9. Is the scale-up a horizontal or vertical scale-up?
10. Are there any risks, fears or weaknesses?
11. *Could you tell me a little bit about the Principal Investigator/s?*
12. What expertise do they bring?
13. How well do you know them? Have you worked with them previously?
14. Does it make a difference if you know them well or not? Why?
15. How do you plan to keep in touch during the process?
16. *Who you think are the relevant stakeholders and end-beneficiaries in your project?*
    1. Have you started consulting with them?
       1. If so, how?
       2. If not, at what stage will you be consulting with them?
    2. What is their role? What are the challenges in ensuring their buy-in?
17. *Could you tell me a little bit about the governance structure of the project team and collaborators?*
18. How is the project structured to enable input from all stakeholders?
19. How do you meet (e.g. Zoom, face-to-face) and how often?
20. How are decisions made and who makes the final decision regarding project processes?
21. *What do you think about the health system in the countries where the project is being implemented? Alternative questions for those who have projects that do not involve the health system: What do you think about the health system and other relevant system in the countries where the project is being implemented?”*
    1. Is it generally open to change and receptive and encouraging to new ideas?
    2. What are they critical challenges that the health system (other system) poses with regard to the project? How do you know this?
    3. How does the project design take into account the health (or other) system challenges?
22. *Are there any industry stakeholders for this project (specific probe if not mentioned in 7 above, or for expansion)?*
    1. Who are they?
    2. What is their role? What are the challenges in ensuring their buy-in?
    3. At what stage will you be consulting with them?
23. *Please tell me a little about the local government and key government stakeholders (specific probe for expansion).*
    1. What is their role and how important is their role?
    2. What do you think are the challenges in partnering with them?
    3. How do you plan to ensure their buy-in and what stage of the project is this important?
    4. Do you see any ongoing issues with regard to this?
24. *Are there any other health service providers who are involved in the delivery of the program (specific probe if not mentioned in 7 above, or for expansion)?*
    1. What is their role?
    2. How do you plan encourage them to participate and contribute?
    3. What are the challenges in keeping them motivated and interested?
25. *Tell me a little about the end-beneficiaries of this program*
    1. What are their health needs currently with regard to hypertension and diabetes?
    2. How are these needs being met currently?
    3. At what stage of the program implementation are they being consulted and in what manner?
    4. Why do you think they will adopt and continue with the model/innovation?
    5. Has the same group of beneficiaries successfully taken up any program previously? Is there a history of acceptance?
    6. What do you perceive to be the main challenges in their uptake of the program?
    7. How do you plan to deal with these challenges?
26. *Tell me a little bit about the front-line staff who will be delivering the program?*
    1. How important do you think their role is?
    2. At what stage of the program is their role most important?
    3. Do they have other conflicting projects or programs that they work in?
    4. What are their challenges and motivators to promote this programme?
    5. What is the incentive for them to promote this program?
    6. What sort of resources are being invested in their training? Is there written documentation of all policies that they need to follow?
    7. Has any training material been prepared for their work?
27. *Has a pilot been conducted? If yes:*
    1. When was the pilot conducted? Where was it conducted?
    2. Was it in the same location as the scale up? (same local environment or different?)
    3. Was the pilot evaluated? If so, what documentation and methods were used to gather data?
    4. How did you or the team assess that the pilot program could be scaled up? How do you know that the program is effective (that it works)? How do you know that the program is an efficient way of delivering to the community?
    5. What were some of the challenges that the team faced during pilot stage? How did you deal with them?
    6. What were the main learnings from the pilot? Do you think that the pilot provided any tweaking to the original program strategy?
    7. What were the learnings about stakeholders- motivators, buy-in, challenges/ barriers?
    8. What were the challenges that arose from the health system perspective?
    9. Has the intervention changed because of the results of the pilot?
    10. How are you measuring fidelity?

*If no pilot was conducted:*

1. *Could you please explain why a pilot or feasibility study was not conducted?*
2. How does the team plan to scope the study, identify local challenges and barriers, and determine what works well and what does not work well?
3. *Please tell me a little bit about the scale-up plan and strategy*
4. Is there a specific plan or strategy that is going to be used to guide the process? Has it been documented?
5. Which members of the team, partners and other stakeholders are aware of this strategy?
6. Which members of the team, partners and other stakeholders have been consulted while drawing this up?
7. Do you see any potential issues for long term sustainability of the program?
8. What do you think will happen if a new unplanned challenge emerges during implementation? What qualities do you think your team or organisation has to cope with this? How will the team know and respond?
9. Are you measuring cost-effectiveness? If so could you briefly explain how?

**Appendix 5:** **Timepoint 0 (T_0_)- Baseline Interview guide for local government stakeholders**

**___________________________________________________________________**

Thank you for agreeing to take part in this interview. My name is X and I am undertaking this interview as a part of my PhD Research the title of which is “Applying a systems lens to identify challenges, enablers and barriers to the GACD scale up interventions”. My supervisors for the PhD research are Prof. Y from University 1 and A/Prof Z from University 2.

The aim of the interview is to collect baseline information about your scale up project.

1. *Can you tell me a little bit about yourself and your motivations that made you to partner in this scale-up project*
2. *Please tell me a little about the scale-up project?*
3. Can you explain briefly what is being scaled-up?
4. What was the opportunity to scale up? Why?
5. Is there anything new or novel about what is being scaled-up?
   1. If it is new, what is new about it (e.g. a technical innovation/process (new delivery approach), organizational innovation (new partnerships), or a combination)?
6. What was the architecture for this scaling opportunity?
7. Who were the actors and players?
8. As a government representative do you think the scale-up is a fairly simple model or is it complex? Why?
9. Are there any risks, fears or weaknesses?
10. *Please tell me a little bit about your government’s role in this project?*
11. Why does this project interest your local government?
12. What is your governments role in this project?
13. In your opinion what are the main strengths that your local government brings?
14. What are the challenges that you think the government systems will face during the scale-up process?
15. *Could you tell me a little bit about the local organisation in the scale-up?*
16. What expertise do they bring?
17. How well do you know them? Have you worked with them previously?
18. Does it make a difference if you know them well or not? Why?
19. How do you, plan to keep with the team during the process? What challenges do you see in keeping this relationship through the process?
20. *When were you first informed about this project? What were you told? Who introduced this project to you?*
21. *Could you tell me a little bit about the governance structure of the project team and collaborators?*
22. How is the project structured to enable input from all stakeholders?
23. How do you meet (e.g. Zoom, face-to-face) and how often?
24. How are decisions made and who makes the final decision regarding project processes?
25. *What do you think about the health system in your country?*
    1. *Is it generally open to change and receptive and encouraging to new ideas?*
    2. *What are they critical challenges that the health system poses with regard to the project? How do you know this?*
    3. *How does the project take into account the health system challenges?*
26. *Tell me a little about the end-beneficiaries of this program*
    1. What are their health needs currently with regard to hypertension and diabetes?
    2. How are these needs being met currently?
    3. At what stage of the program implementation are they being consulted and in what manner?
    4. Why do you think they will adopt and continue with the model/innovation?
    5. Has the same group of beneficiaries successfully taken up any program previously? Is there a history of acceptance?
    6. What do you perceive to be the main challenges in their uptake of the program?
    7. How do you plan to deal with these challenges?
27. *Were you involved in a pilot of this project? If yes:*
    1. How did you assess that the pilot program could be scaled up? How do you know that the program is effective (that it works)? How do you know that the program is an efficient way of delivering to the community?
    2. What were some of the challenges that your local government faced during pilot stage? How did you deal with them?
    3. What were the main learnings from the pilot? Do you think that the pilot provided any tweaking to the original program strategy?
    4. What were the challenges that arose from the health system perspective?
    5. Has the intervention changed because of the results of the pilot?
28. *Please tell me a little bit about the scale-up plan and strategy*
29. Is there a specific plan or strategy that is going to be used to guide the process? Has it been documented?
30. Were you consulted in this process?
31. Do you see any potential issues for long term sustainability of the program?
32. What do you think will happen if a new unplanned challenge emerges during implementation? What qualities do you think your local government has to cope with this?

**Appendix 6: Timepoint 0 (T_0_) Baseline Interview front-line workers and staff**

**_________________________________________________________________**

Thank you for agreeing to take part in this interview. My name is X and I am undertaking this interview as a part of my PhD Research the title of which is “Applying a systems lens to identify challenges, enablers and barriers to the GACD scale up interventions”. My supervisors for the PhD research are Prof. Y from University 1 and A/Prof Z from University 2.

1. *Tell me little bit about the community and end-beneficiaries of this project:*
   1. What is their general awareness level about hypertension and diabetes?
   2. What are their health needs currently with regard to hypertension and diabetes?
   3. How are these needs being met currently?
   4. Were they involved during or are they being consulted and in what manner?
   5. Why do you think they will adopt and continue with the model/innovation?
   6. Has the same group of beneficiaries successfully taken up any program previously? Is there a history of acceptance?
   7. What do you perceive to be the main challenges in their uptake of the program?
2. *Tell me about your role and work on this project?*
   1. Who is your employer?
   2. What are your main responsibilities?
   3. When did you start working in this role?
   4. Why did you take up this job?
   5. What do you think will help you to do this role well?
   6. What do you think is the most challenging or difficult thing about your role?
3. *Do you have any other similar projects or programs that you work for presently?*
   1. Are these roles similar or different? In what way?
   2. Do you think this be a challenge for you to balance all these roles?
4. *About governance systems:*
   1. Who do you report to and how do you report to them?
   2. What supports do you receive from the organisation?
   3. If you have a problem with regard to this project- what will you do? Who will you contact?
5. *Do you think Covid-19 might have an impact on your job in anyway?*
   1. How?
   2. Did you have to change your working in any manner?
