# Supplementary material for: Strengthening policy engagement when scaling up interventions targeting non-communicable diseases: insights from a qualitative study across 20 countries
Source: Health Policy Plan. 2024 Nov 18;39(Suppl 2):i39–53. doi: 10.1093/heapol/czae043 (PMC11570794; doi:10.1093/heapol/czae043)
Supplement: czae043_Supp [file czae043_supp.zip › suppl_data/Appendix 1. List of funded scale-up studies.docx]

**Appendix 1: List of scale-up projects funded in the GACD scale-up call in 2019**

| **Name** | **Location of scale-up (country)** | **Programme contact** |
| --- | --- | --- |
| 1. DIABFRIL - LATAM | Argentina, Chile, Columbia, Mexico, Peru | Leocadio Rodriguez Manas |
| 1. SUNI-SEA - Scaling-up NCD Interventions in South East Asia | Indonesia, Myanmar, Vietnam | Maarten Postma |
| 1. SCUBY | Belgium, Cambodia, Slovenia | Josefien van Olmen |
| 1. WHO PEN at Scale | Eswatini | Jan-Walter De Neve |
| 1. INTE-AFRICA | Tanzania, Uganda | Shabbar Jaffar |
| 1. Evaluating the role of pharmacists and m-Health strategies in the management of hypertension in General Pueyrredon | Argentina | Maria Eugenia Esandi |
| 1. Implementation of a model of management of myocardial infarction patients. | Belgium | Carlos Daniel Tajer |
| 1. Healthy Schools | Argentina | Jonatan Konfino |
| 1. Use of mHealth and social media to strengthen a primary prevention program for type2 diabetes in public primary care clinics | Argentina | Andrea Beratarrechea |
| 1. Evaluating and bringing to scale alternative food networks to address diabetes mellitus and hypertension | Ecuador | Malek Batal |
| 1. Scaling up the Community Health Assessment Program in the Philippines (CHAP-P) | Philippines | Gina Agarwal |
| 1. Community mHealth Integrated Care (ComHIC) to manage hypertension/diabetes in Tanzania’s overburdened health system | Tanzania | Keiko Nakamura |
| 1. Community-based lifestyle intervention for diabetes management in rural Nepal | Nepal | Tomohiko Sugishita |
| 1. School-based education programme to reduce salt: Scaling up in China (EduSalts) | China | Feng He |
| 1. Scaling up the Primary Health Integrated Care Project for Chronic Conditions in Kenya an implementation research project | Kenya | Pablo Perel |
| 1. The Bangladesh D:CLARE Project [Diabetes: Community-Led Awareness, Response and Evaluation] | Bangladesh | Ed Fottrel |

| **Name** | **Location of scale-up (country)** | **Programme contact** |
| --- | --- | --- |
| 1. CHArMING - Control of Hypertension and diAbetes in MINas Gerais | Brazil | James Batchelor |
| 1. Scaling up food policy interventions to reduce non-communicable diseases in the Pacific Islands | Fiji, Samoa | Jacqui Webster |
| 1. An evaluation of the Resolve to Save Lives salt reduction program in China | China | Bruce Neal |
| 1. Strengthening China’s essential public health package for hypertension and diabetes care in rural village clinics through meaningful use of health information systems | China | David Peiris |
| 1. Scale-up of a primary care intervention for cardiovascular risk management in Malang, Indonesia | Indonesia | Anushka Patel |
| 1. Scaling up interventions to improve the control of hypertension and diabetes in partnership with the governments of Kerala and Tamil Nadu | India | Brian Oldenburg |
| 1. Evaluating the implementation of Group Empowerment and Training (GREAT) for diabetes in South Africa. | South Africa | Robert Mash |
| 1. Assessing COPC scale-up in selected learning sites of the Cape Town Metro: community-based NCD prevention interventions (ACCELERATE) | South Africa | S Read |
| 1. Evaluating the implementation and scale-up of Nigeria National Salt reduction program | Nigeria | Dike Bevis Ojji |
| 1. Implementing and scaling up a team-based care strategy for hypertension control in Colombia and Jamaica. | Jamaica and Colombia | Marshall Tulloch-Reid |
| 1. Addressing hypertension and diabetes through community engaged systems in Puno, Peru (Andes study) | Peru | German Malaga |

**Appendix 1: List of scale-up projects funded in the GACD scale-up call in 2019**

(Available from <https://www.gacd.org/research/projects>)

*Please note: This table was first published as a Supplementary Table in Ramani-Chander A, Joshi R, van Olmen J, Wouters E, Delobelle P, Vedanthan R, Miranda JJ, Oldenburg B, Sherwood S, Thrift AG, on behalf of the Global Alliance from Chronic Diseases Upscaling Working Group Collaborators.**Applying Systems Thinking to Identify Enablers and Challenges to Scale Up of Interventions for Diabetes and Hypertension in Low- and Middle-Income Countries: Protocol for a Longitudinal Mixed-Methods Study. BMJ Open 12:e053122. DOI:*[*https://doi.org/10.1136/bmjopen-2021-053122*](https://doi.org/10.1136/bmjopen-2021-053122)*. Used with permission.*
