# Supplementary material for: Strengthening policy engagement when scaling up interventions targeting non-communicable diseases: insights from a qualitative study across 20 countries
Source: Health Policy Plan. 2024 Nov 18;39(Suppl 2):i39–53. doi: 10.1093/heapol/czae043 (PMC11570794; doi:10.1093/heapol/czae043)
Supplement: czae043_Supp [file czae043_supp.zip › suppl_data/Appendix 2. Detailed Methods (amended).docx]

**Appendix 2: Detailed Methods**

*Study setting*

This study forms part of the joint research activities undertaken by the GACD Upscaling Working Group Collaboration, a group that includes researchers from the funded studies and other researchers from within the GACD network. The protocol for the joint research has been described.(Ramani-Chander *et al.*, 2022)

The funded studies were diverse in terms of country of implementation, approaches adopted, and on how the research was planned.(Ramani-Chander *et al*, 2023) The interventions also varied and included health apps for screening, integration of services for delivery of NCD care, provision of locally relevant WHO packages of interventions, strengthening alternate food networks, or education of communities about NCDs.

One of the GACD priorities for the scale-up funding call was to engage with policymakers.(Global Alliance for Chronic Diseases, 2019) There was evidence of planned policy engagement strategies, such as including policymakers in the grant applications, involving them during steering meetings, and taking their advice for site selection. However, details of policy engagement planning processes varied with some using specific frameworks to guide the process.(Ramani-Chander *et al.*, 2023)

*Study design*

This qualitative study is focused on understanding the policy engagement experiences of stakeholders during the early implementation phase of these funded scale-up research projects. Stakeholders in a process refer to the actors (persons or organizations) with a vested interest in the policy being implemented.(Schmeer, 2000 #645) For this study we identified four groups of stakeholders:

(1) *Principal Investigators (PIs) and Co-PIs* who had oversight and overall responsibility for the research planning and implementation;

(2) *Other Project Investigators* who led particular components of the research such as stakeholder engagement or process evaluation;

(3) *Project Implementers*, who led the work on the ground, and staff members, who worked for the project in different capacities.

(4) *Government representatives*, who represent federal and regional policymaking in the implementing country.

To elicit the perceptions of stakeholders, the working group members collaboratively developed four semi-structured interview guides in English for each relevant stakeholder group (Appendices 3 to 6). Interview guides were deductively developed by identifying common elements (including characteristics of the intervention, importance of pilot phase, and stakeholder engagement processes) featured in eight frameworks designed to facilitate the scale-up of complex health interventions including ExpandNet. (Barker *et al.*, 2016; Bradley *et al.*, 2012; Chambers *et al.*, 2013; Cooley and Linn, 2014; Greenhalgh *et al.*, 2017; Milat AJ, 2014; World Health Organization, 2009; Yamey, 2011) The interview guides addressed a range of issues related to scaling up, and these broader findings have been reported separately. (Ramani-Chander *et al.*, in press, 2024) In this study, we focus on presenting the policy engagement experiences and activities in detail as this is a vital component in the scale-up process for NCD-related interventions. The specific questions in the interview guides (Appendices 3 to 6) that provided deeper insights into the policy engagement activities have been highlighted in blue text. These included process of identification and role of government stakeholders in the research process, method of communication, time of engagement, and questions addressing system level challenges and facilitators.

*Data collection*

Each of the scale-up research consortia included a multidisciplinary team of investigators representing both the high-income (HIC) and the implementation countries, the latter primarily being LMICs. Research activities were conducted collaboratively across teams of researchers from all countries. The GACD secretariat provided details of the PIs who served as the primary contacts for the study team. PIs from all 27 funded studies were invited to be interviewed and were asked to facilitate contact with other team members who would provide a mix of stakeholders to be interviewed. Researcher X completed the recruitment process with due ethical diligence, and conducted all interviews using Zoom (Zoom Video Communication, Inc., San Francisco, USA). Interviews were conducted between August 2020 and July 2021. The interviews were audio recorded and then transcribed verbatim using a professional transcription service. Researcher Y and Researcher Z were closely involved in all aspects of data collection. They were systematically debriefed after each interview, enabling reflection on the data obtained and refinement of follow-up questions with other project stakeholders. (McMahon and Winch, 2018)

*Data analysis*

The core research team for this study comprised Researcher X, Y, and Z who were a part of the working group collaboration but external to any of the funded scale-up projects thereby providing an independent perspective for conducting this study. The data pertaining to this study, including participant details recordings, transcripts, thematic coding analysis were accessible to the core research team only. All other co-authors did not have access to specific data pertaining to the study. We undertook inductive, open coding using thematic analysis (Braun and Clarke, 2006; Braun and Clarke, 2021) to help retain the richness of data obtained, facilitated by NVivo software (QSR International Pty. Ltd., Version 12). Thematic analysis was defined as a “method for identifying, analyzing and reporting patterns (themes) within data” (Braun & Clarke, 2008) and provided flexibility to analyse the data without being prescriptive about the generated patterns or themes. Researcher X conducted the coding process and Researcher Z independently coded 10% of transcripts. The core group worked closely engaging in weekly discussions to analyse the findings until a common understanding of the codes was achieved.(Joshi et al., 2009; Keene; O’Connor & Joffe, 2020) This collaborative process helped in the interpretation of data and was continued until all transcripts were coded. Through this process, codes were collapsed or expanded and coding labels were refined. At the end of the coding process, 63 codes generated 15 themes which reflected patterns of meaning shared by the participants. These included both challenges and facilitators faced during the process of policy engagement. We present findings in a deidentified manner with no reference to the participant or the project they represented.
